# Medical Plant Extract Purification from Cadmium(II) Using Modified Thermoplastic Starch and Ion Exchangers

**DOI:** 10.3390/ma14164734

**Published:** 2021-08-22

**Authors:** Yi-Gong Chen, Qian Wang, Anna Wołowicz, Agnieszka Gładysz-Płaska, Monika Wawrzkiewicz, Weronika Sofińska-Chmiel, Gui-Yuan Lv, Dorota Kołodyńska, Su-Hong Chen

**Affiliations:** 1 Collaborative Innovation Center of Yangtze River Delta Region Green Pharmaceuticals, Zhejiang University of Technology, Chaowang Road 18, Hangzhou 310014, China; yigongchen@hotmail.com; 2 Hangzhou Fang Hui Chun Tang Group Co., Ltd., Hangzhou 310052, China; wangq@fhct.com; 3 Department of Inorganic Chemistry, Institute of Chemical Sciences, Faculty of Chemistry, Maria Curie-Skłodowska University, M. Curie Skłodowska Sq. 2, 20-031 Lublin, Poland; anna.wolowicz@poczta.umcs.lublin.pl (A.W.); a.gladysz-plaska@poczta.umcs.lublin.pl (A.G.-P.); m.wawrzkiewicz@poczta.umcs.lublin.pl (M.W.); 4 Analytical Laboratory, Institute of Chemical Sciences, Faculty of Chemistry, Maria Curie-Skłodowska University, M. Curie Skłodowska Sq. 2, 20-031 Lublin, Poland; wschmiel@poczta.umcs.lublin.pl; 5 College of Pharmaceutical Science, Zhejiang Chinese Medical University, Hangzhou 310053, China; zjtcmlgy@163.com

**Keywords:** cadmium, *Dendrobium officinale Kimura* et Migo, thermoplastic starch, ion exchange resin, plant extract purification

## Abstract

Pure compounds extracted and purified from medical plants are crucial for preparation of the herbal products applied in many countries as drugs for the treatment of diseases all over the world. Such products should be free from toxic heavy metals; therefore, their elimination or removal in all steps of production is very important. Hence, the purpose of this paper was purification of an extract obtained from *Dendrobium officinale Kimura* et Migo and cadmium removal using thermoplastic starch (S1), modified TPS with poly (butylene succinate); 25% of TPS + 75% PBS (S2); 50% of TPS + 50% PLA (S3); and 50% of TPS + 50% PLA with 5% of hemp fibers (S4), as well as ion exchangers of different types, e.g., Lewatit SP112, Purolite S940, Amberlite IRC747, Amberlite IRC748, Amberlite IRC718, Lewatit TP207, Lewatit TP208, and Purolite S930. This extract is used in cancer treatment in traditional Chinese medicine (TCM). Attenuated total reflectance-Fourier transform infrared spectroscopy, thermogravimetric analysis with differential scanning calorimetry, X-ray powder diffraction, gel permeation chromatography, surface analysis, scanning electron microscopy with energy dispersive X-ray spectroscopy, and point of zero charge analysis were used for sorbent and adsorption process characterization, as well as for explanation of the Cd(II) sorption mechanism.

## 1. Introduction

Medicinal plants, being a part of conventional medicine, have been used in therapy throughout the world for a long time. They have been trusted globally for thousands of years for their accessibility and limited side-effects [1]. The World Health Organization (WHO) estimates that 65–80% of the world’s population, especially in Africa, Asia, Latin America, and the Middle East use herbal products [2]. Recently, greater attention has also been paid to herbal medicine in developed countries. The methods generally called natural medicine, based on herbal extracts, are not favored [3,4,5,6]. In fact, these are proven and non-contradictory to conventional medicine. They are not only a ‘green’ alternative to most pharmaceuticals used for the treatment of diseases all over the world, but also the source of a wide variety of natural antioxidants. Their medicinal value is usually due to the presence of very important phytochemicals, such as alkaloids and tannins, as well as flavonoids and phenolic compounds. However, they should be free from heavy metal ions. For this purpose, the key to success is the control of the process from the beginning (cultivation) to the end (production cycle) and quality assurance, throughout the complete process chain. Therefore, the critical factors for the manufacturing of extracts are the raw vegetal material, manufacturing process, and final adjustment by blending.

**Figure 1:**
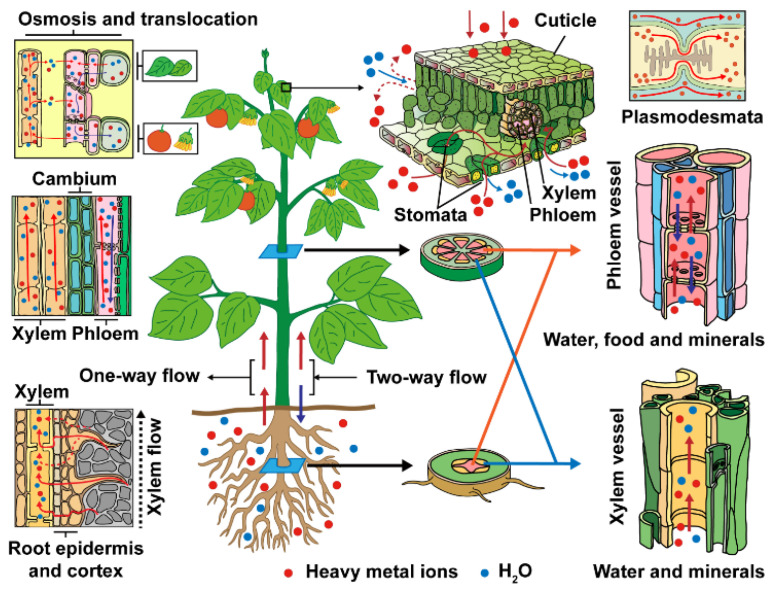
A schematic model for translocation of heavy metal ions in plants.

Excess amounts of both essential and non-essential metals can cause ion stress in plants and lead to a variety of direct or indirect effects, which involve virtually all physiological functions. Heavy metal ions are distributed in growth substrates, widely transported to various organs of plants, and then enter the food chain. Trace metal ions are contained in growth substrates, especially the soil. Due to the non-degradability and cytogenetic effects of heavy metals, biological structures, biochemical, physiological, and metabolic processes suffer from sustainable irreversible damage, and disturb or even collapse biological systems by inducing changes at the transcription level of numerous protein-encoding genes, which cause chlorosis, growth retardation, induce lipid peroxidation, photosynthesis inhibition, enhanced proteolysis, disorder reactive oxygen species (ROS) and antioxidants, and eventually apoptosis or cell death. To discover the mechanism of translocation and accumulation in detail, it is essential to study the processes of heavy metal ion uptake in plants. Metal mobilization, root uptake, compartmentation, sequestration, xylem loading, distribution in shoots, and storage in leaf cells are the main steps involved in translocation and accumulation of heavy metals in plants (Figure 1). 

**Figure 2:**
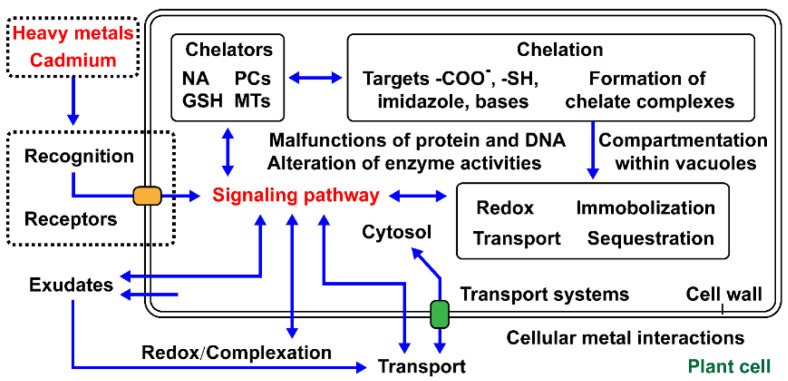
Some important aspects of cellular heavy metal interactions in plants.

The nutrients required by plants in the greatest amounts are N, P, and K. For this reason, they are often considered the most important; N and P as components of proteins and nucleic acids. The essential heavy metals, such as Fe(II), Cu(II), Zn(II), Ni(II), Co(II), Mo(II), Mn(II), and Se(IV,VI), play a crucial regulatory role in the series of cellular reactions, including electron transfer, enzyme activation, redox reactions, and pigment synthesis. However, Pb(II), Cr(III), Cd(II), Hg(II), and As(V) are not required in any biological reactions and cause toxic effects [3]. Except for the physiological defensive mechanisms against damage by heavy metals (sequestration, compartmentalization, exclusion, and inactivation), plants can also induce antioxidant systems and maintain metal homeostasis by limiting the bioavailability of metals [4]. The essential processes: uptake, translocation, detoxification, and accumulation of heavy metals are controlled by physiological and molecular complex regulatory mechanisms (Figure 2). For instance, transporters for Zn(II) and Ca(II) can take up Cd(II) [6], and transporters for phosphate can also absorb arsenate [7].

**Figure 3:**
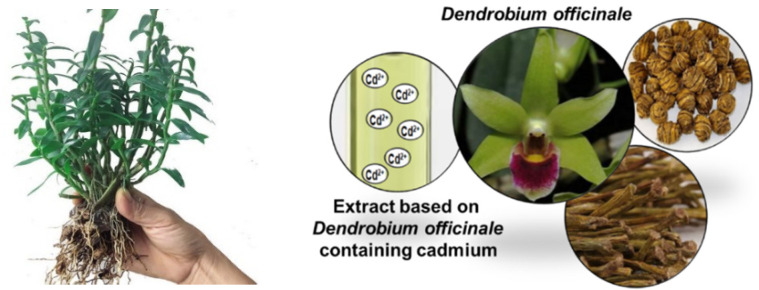
*D. officinale* parts typically used for obtaining extracts in THM.

As for the transport of metal ions in plants, both apoplastic and symplastic pathways can be distinguished. Heavy metals using the apoplastic pathway can reach the apical region of the underdeveloped endodermal suberin lamellae [8]. The translocation and accumulation processes are closely related to the mobilization, radial transport [9], and long-distance transport in xylem, as well as sequestration and detoxification of metal ions [10]. Radial translocation, in particular, is essential for the accumulation of heavy metals in the above-ground organs. For example, Pb(II) in the epidermal and cortical cells of the roots cannot be loaded to the steles, which leads to a large accumulation in the roots that cannot be further transported to the shoots driven by the transpiration stream [11,12]. Similarly, this effect was observed in Cr-tolerant plants. The Cr absorbed by Typha angustifolia is mainly distributed in the outer layer of the roots, while only a minor portion of Cr is transferred to fronds and distributed uniformly [13]. The method of transport and place where heavy metal ions accumulate (roots, leaves, steams, blossoms) play a significant role when considering the part of the plant from which the extract is obtained, especially in traditional herbal medicine (THM). These aspects are presented based on the *Dendrobium officinale Kimura* et Migo used in this study (Figure 3). *D. officinale* is a famous Chinese plant used in traditional Chinese medicine (TCM). Its extracts contain various phytochemicals, particularly polysaccharides, that have nutraceutical and pharmaceutical value. They are commonly used to reduce blood lipids, promote blood circulation, and improve body immunity. They are also very important during radiotherapy and chemotherapy to reduce the induced side effects (enhance suppression and resistance to free radical damage), improve quality of life, and survival time during cancer treatment. 

However, in the case of hyperaccumulators, metal ion transport occurs by bulk flow in the xylem from root to shoot by complex formation with chelators [14]. Heavy metal complexes are translocated to the neighboring cells step by step by plasmodesmata or transporters on the plasma membrane from the root symplast into the xylem apoplast. In this saturable process, the number of transport proteins (Table 1), as well as the rate of transport, substrate affinity, and substrate specificity of variation in the transporters, are the main limiting factors [15].

Subsequently, metals are transported from the xylem into the leaf cells. Heavy metals are typically bound by chelators and tend to concentrate in the epidermis and trichomes at the tissue level [32,33], while the excess parts accumulate in vacuoles or cell walls at the cellular level [34,35]. They are probably accumulated in the plants by a double use of the transport system [36]. During the translocation process of Cd(II) from the root to xylem, the Zn(II) active transport system components that involve heavy metal ATPase are used. The typical effects of Cd(II) are the inhibition of mitosis and repression of auxin production and signaling [37]. By the disorganization of microtubule, cytoskeleton, and tubulin structures, Cd(II) can damage meristems. Furthermore, Cd(II) is translocated in the xylem mainly as aqueous free ions instead of being complexed with citrate. After going through the uptake, transport, sequestration, and detoxification processes of herbaceous and woody plants, Cd(II) is partially translocated to the xylem vessels from roots and sequestered and detoxified in the vacuoles of cells [38]. Inadequate methods of manufacturing, as well as unhygienic processing and packing, are the major sources of microbial contamination in herbal medicine products. This could be a major source of metal contamination in herbal medicine products.

The aim of this paper was to investigate the possibility of the purification of Chinese herbal medicinal plant extracts, based on *D. officinale*, from Cd(II), as well as to investigate the sorption properties of various forms of modified thermoplastic starch (TPS) as well as commercially available ion exchangers (IXs). Moreover to date, there are no data in the literature that compare these two kinds of sorbents. Generally *D. officinale* has anti-oxidation, anti-fatigue, and anti-aging effects for the human body. Therefore it is very important to obtain pure extracts without Cd(II) ions. Different materials, both of a natural origin and commercially available, were proposed for purification.

The TPS sorbents were obtained from potato starch containing 20% amyloze with glycerine 99.5% (samples denoted as S1, S2, S3, and S4), whereas ion exchange resins (denoted as SP112, S940, IRC747, IRC748, IRC718, TP207, TP 208, and S930) were chosen based on the producer data sheets, their properties, functional groups, and application area. Previous studies found that the ion exchangers are useful for heavy metal ion removal from wastewaters [39,40,41,42]. To characterize the TPS sorbents, the following methods were used: attenuated total reflectance-Fourier transform infrared spectroscopy (ATR-FTIR), thermogravimetric analysis (TGA) with differential scanning calorimetry (DSC), X-ray powder diffraction (XRD), and gel permeation chromatography (GPC). Moreover, Brunauer–Emmett–Teller (BET) surface analysis, scanning electron microscopy with energy dispersive X-ray spectroscopy (SEM-EDX), point of zero charge analysis (pH_pzc_ analysis), and elemental analysis (CHN) were used for sorbents and adsorption process characterization, as well as for explanation of the sorption mechanisms. The effects of pH, initial Cd(II) concentration, and phase contact time were taken into account during the extract purification. The kinetic studies were analyzed with the typical kinetic models, such as pseudo-first order and pseudo-second order, as well as intraparticle diffusion. The maximum adsorption capacities were obtained, and the Langmuir and Freundlich isotherm models were applied. The most effective methods for purification of *D. officinale* extracts were discovered.

## 2. Materials and Methods

### 2.1. Sorbents and Ion Exchangers

The TPS sorbents were obtained from the potato starch containing 20% amyloze (Trzemeszno, Polska), with the addition of 99.5% plant pharmaceutical glycerol and water as plasticizers. 

The procedure of TPS production is presented in [43,44]. Three sorbents, obtained on the basis of TPS starch, were used in the research and were determined successively: TPS, sorbent 1 (S1) containing starch, 30% glycerol and 8% of water (used for comparison); sorbent 2 (S2), obtained by mixing 25% TPS + 75% PBS (poly(butylene succinate)); sorbent 3 (S3), obtained by mixing 50% TPS + 50% PLA (polylactic acid); and sorbent 4 (S4), obtained by mixing 50% TPS + 50% PLA and additionally 5% hemp fibers (Table 2). A comparison of PBS and PLA is presented in Table S1 (Supplementary Materials).

As for the ion exchangers (IX), the following were applied: Lewatit MonoPlus SP112 (denoted as SP112), Purolite S940 (denoted as S940), Amberlite IRC747 (denoted as IRC747), Amberlite IRC 748 (denoted as IRC748), Amberlite IRC718 (denoted as IRC 718), Lewatit TP207 (denoted as TP207), Lewatit TP208 (denoted as TP208), and Purolite S930 (denoted as S930). The characteristics of these chelating ion exchangers are presented in Table 3 (functional groups, appearance) and in Table S2 (Supplementary Materials).

### 2.2. Instruments

Fourier transform infrared spectroscopy (ATR-FTIR) can be used as a method of quick and effective identification of changes in sorbent properties. This was used for detection of the chemical bonds in the samples. Therefore, a Cary 630 ATR-FTIR spectrometer with the attenuated total reflectance mode (Agilent Technologies, Santa Clara, CA, USA) was used. The spectra were recorded without any sample preparation in the range 4000–650 cm^−1^.

The analyses were performed using a TA Instruments Q50. The test sample, weighing about 10 mg was placed on a platinum measuring pan and then in a TGA furnace. It was heated at a constant rate of 10 °C/min in a temperature range of 298–723 K, and the change in mass as a function of temperature was recorded. 

The XRD measurements were carried out using an X’pert MPD X-ray diffractometer (Panalytical, Eindhoven, Netherlands) with a goniometer PW 3020 and X-ray source anode Cu (K_α_) (I = 40 mA and U = 40 kV) and a graphite monochromator. Diffraction patterns were recorded, and HighScore Pro software (version 3.0, Eindhoven, The Netherlands) was used for diffraction data processing. The identification of mineral phases was based on PCPDFWIN ver. 1.30 formalized by JCPDS-ICDD. 

Accelerated surface area and porosimetry analysis using ASAP 2045 (Micromeritics, Inc., Norcross, GA, USA) with Brunauer–Emmett–Teller (BET) analysis was also performed. nitrogen adsorption–desorption analysis was performed at 77 K.

Scanning electron microscopy with energy dispersive X-ray spectroscopy (SEM-EDAX) (Tescan, Fuveau, France) was also used to compare the sorptive material beads.

The point of zero charge (pH_pzc_) of the S1–S4 samples and ion exchangers SP112, S940, IRC747, IRC748, IRC718, TP207, TP207, TP208, and S930 was measured using the pH drift method using a pH-meter CP-411 (Elmetron, Zabrze, Poland). 

All these methods were used for sorbent and adsorption process characterization, as well as for explanation of the sorption mechanisms. In the second step, the sorption properties were studied with respect to the effect of the pH, the comparison of uptake kinetics (at different levels of saturation of the sorbent), and the evaluation of sorption isotherms.

### 2.3. Methods; Kinetic and Adsorption Experiments

All sorptive materials were used to remove Cd(II) ions from the *D. officinale* extracts. The study of sorption properties was carried out in batch mode; 0.2 g of sorbent was mixed with 20 cm^3^ of solution containing Cd(II) ions at the above-mentioned initial concentration [C_0_, mg/dm^3^] at the established pH. The pH measurements were performed using a pH-meter, CP-411 (Elmetron, Zabrze, Poland). A laboratory shaker, type 358A (Elpin+, Lubawa, Poland) at amplitude 8 and stirring rate 180 rpm, was used for the batch experiments. The sorbent dosage was defined as m/V. The standard temperature was 295 K. The samples of solutions were collected and filtrated, and the residual concentration of Cd(II) was measured using an atomic absorption spectroscopy method (AAS). A Spectr AA240 FS atomic absorption spectrophotometer (AAS, Varian Inc., Melbourne, Australia), operating with an air-acetylene flame, was used to analyze the concentration of Cd(II) ions.

Kinetic experiments and the study of pH value effect were carried out at the initial Cd(II) concentrations of 10 mg/dm^3^, 50 mg/dm^3^, and 100 mg/dm^3^ for S1–S4 and 10 mg/dm^3^, 25 mg/dm^3^, and 50 mg/dm^3^ for IX. The effect of pH was determined by studying the adsorption of Cd(II) ions over a pH range 2–10. The pH was adjusted by the addition of HCl or NaOH solution using a pH-meter, CP-411 (Elmetron, Zabrze, Poland). All the experimental data were the averages of triplicate determinations. The relative errors of the data were about 5%.

Typical kinetic models were used for fitting experimental results, i.e., pseudo-first (PFO) and pseudo-second order (PSO), intraparticle diffusion equations (IPD), and sorption isotherms, i.e., Freundlich and Langmuir. The parameters were determined by non-linear regression analysis using Origin software. The fitting of experimental profiles was compared based on the determination coefficients calculated through the linear regression analysis. The relevant equations are reported in the Supplementary Materials (Table S3).

### 2.4. Calculations

The amount of Cd(II) adsorbed on the thermoplastic starch and the selected ion exchangers (*q_t_*) [mg/g], the amount of Cd(II) adsorbed on the starch, the selected ion exchangers equilibrium capacity (*q_e_*) [mg/g], the sorption %*S,* and desorption %*D* percentages, as well as the distribution coefficient (*K_d_*) [cm^3^/g], were calculated from the difference between the initial and equilibrium concentrations using the equations:

(1) $$q_{t}=\left(C_{0}-C_{t}\right)\times \frac{V}{m}$$

(2) $$q_{e}=\left(C_{0}-C_{e}\right)\times \frac{V}{m}$$

(3) $$\%S=\frac{\left(C_{0}-C_{t}\right)}{C_{0}}\times 100\%$$

(4) $$\%D=\frac{C_{des}}{C_{0}}\times 100\%$$

(5) $$K_{d}=\frac{\left(C_{0}-C_{t}\right)}{C_{t}}\times \frac{V}{m}$$

where *C*_0_ is the initial Cd(II) concentration [mg/dm^3^], *C_t_* is Cd(II) concentration after time *t* [mg/dm^3^], *m* is the thermoplastic starch mass or the ion exchanger mass [g], *V* is the volume of the solution [dm^3^], and *C_des_* is Cd(II) concentration after the desorption [mg/dm^3^]. 

## 3. Results and Discussion

### 3.1. Chemical Characterization of the Materials

Starch is one of the most promising polymers obtained from renewable sources, and which can be successfully used in the biodegradable plastic industry. The glass transition temperature (503 K) and the melting point (493–513 K) of native starch are higher than the degradation temperature (approx. 493 K), therefore starch in its pure form cannot be processed. To obtain a material that can be processed by classical processing methods, so called thermoplastic starch (TPS) must be obtained in the extrusion process [39,40] using a single-screw or twin-screw extruder. Extrusion is a continuous process that involves plasticizing a polymeric material in a plasticizing system, which is ended by head extrusion, which forms the extruded material. The most commonly used TPS plasticizer is glycerol (S1). This alcohol has three hydroxyl groups in its molecule. During the plasticization process between them and the -OH groups of the starch chain, hydrogen bonds are formed that are more stable than the existing intramolecular and intermolecular hydrogen bonds in the starch chains. Water also plays an important role in plasticizing the native starch, taking part in the process plasticization together with the main plasticizer. In the case of a lack of water, it is necessary to increase the quantity of the plasticizers. Raw TPS can be used in powder, flake, chips granule, and fiber forms. This is a cheap and easily accessible approach.

In our research, as modifiers, poly (butylene succinate) PBS as an aliphatic polyester (obtained on a commercial scale in the polycondensation process of succinic acid and 1,4-butanediol) and polylactic acid (PLA) as biodegradable polymer (belonging to the group of aliphatic polyesters) (S2–S4) were used. A comparison is presented in Table S1. Additionally, sorbent S4 was modified by adding hemp fibers. Hemp fibers are a natural and extremely durable raw material used in many industries. Durability, ecological origin, and the possibility of wide applications are the features that best describe the fiber properties. 

#### 3.1.1. ATR-FTIR Analysis 

**Figure 4:**
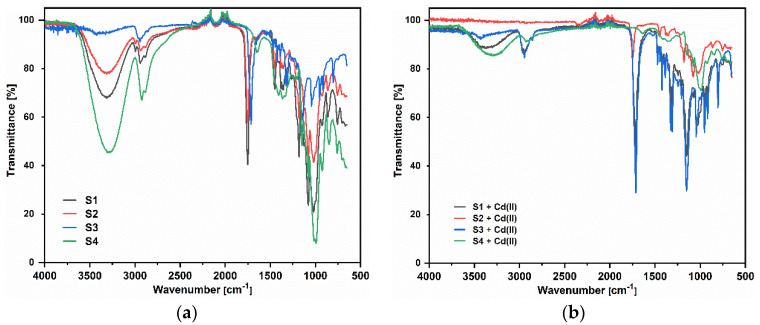
ATR-FTIR spectra of S1-S4, (**a**) before and (**b**) after the sorption process of Cd(II).

Chemical characterization of S1, S2, S3, and S4 samples by the attenuated total reflectance Fourier transform infrared spectroscopy (ATR-FTIR) method was used for assessment of structural changes in the chemical structure of starch before and after the adsorption process. The ATR-FTIR method is also commonly used for the study of structural chemical compounds and sorbent modification. To characterize the S1–S4 sorbents and their structural changes in the chemical structure of starch during the plasticizing process, as well as the extrusion process, at first the ATR-FTIR spectra of native starch (NS), glycerol, PBS, PLA, and TPS were recorded. They are presented in Figure S1. It was proven that, due to the plasticizer addition, shear forces, and temperature during the extrusion process, the intramolecular and intermolecular hydrogen bonds between the hydroxyl groups of the starch chains are broken. These are replaced by more stable hydrogen bonds formed between the -OH groups of the starch and the plasticizer. In the spectrum of NS, the band at the wavenumber of 3303 cm^−1^ coming from hydrogen bonds is shifted to 3287 cm^−1^ in TPS. Characteristic changes in the spectrum of TPS as compared to NS also take place in the region of 900–1200 cm^−1^. These are associated with C-O stretching vibrations in the C-O-H system at 1083 cm^−1^ and 1151 cm^−1^, as well as at 996 cm^−1^, resulting from the stretching vibrations of the C-O bonds in the C-O-C system. Both can form hydrogen bonds with the plasticizer, which results in a shift of these bands to the values 1151 cm^−1^ and 1078 cm^−1^ (Figure S1e) [45]. There is also an additional band next to the wavenumber 1016 cm-1, which, similarly to the wave number 996 cm^−1^, is related to the stretching vibrations of C-O bonds in the C-O-C system. Table 4 shows the changes in the wavenumber values for the characteristic bands of TPS, depending on the modification (Figure 4). The spectra after the sorption of Cd(II) are also presented.

**Figure 5:**
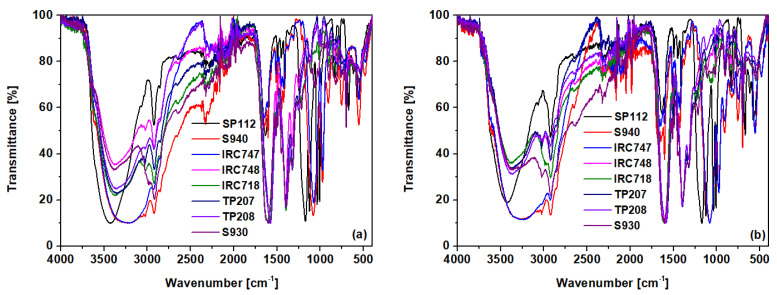
ATR-FTIR spectra of SP112, S940, IRC 747, IRC748, IRC718, TP207, TP208, and S930 (**a**) before and (**b**) after the sorption process of Cd(II).

An ATR-FTIR analysis of the ion exchangers is presented among others in (Figure 5) [46,47]. As for SP112, the bands at 1173, 1124, 1033, and 1009 cm^−1^ before loading are attributed to the presence of sulfonic groups. These bands prove the presence of stretching vibrations of the S=O and S-O groups in the -SO_3_Na group. After sorption, they were moved to 1170, 1120, 1031, and 1001 cm^−1^ for Cd(II) ions. Analogous results were obtained for S940, IRC 747, IRC 748, IRC718, TP207, and TP208. As for S940 and IRC 747, the presence of bands related to P=O stretching vibrations was observed in the range 1350–1150 cm^−1^ and the P-OH group in the range 1100–900 cm^−1^ and for bands in the range 1250–1020 cm^−1^. They are derived from C-N stretching vibrations of aliphatic amines. Next, four ion exchangers, IRC 748, IRC718, TP207, and TP208, contain the iminodiacetate functional groups.

#### 3.1.2. TGA Analysis 

**Figure 6:**
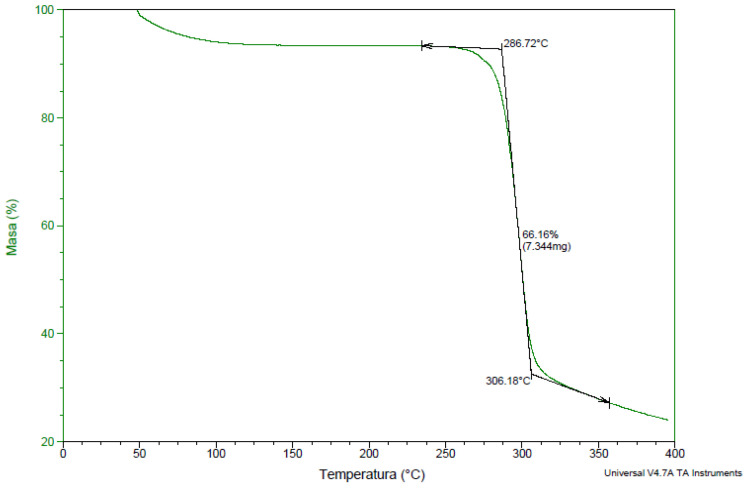
TG curve of S1.

Thermogravimetric analysis (TGA) was used to determine the composition of materials, thermal stability, and temperature degradation [48]. On the TG curve of the granulate of native starch plasticized with glycerol (Figure 6), the first weight loss of 10–15% up to the temperature of 290 °C is related to evaporation of water and plasticizer. The starting temperature for the actual starch-related transformation is 290 °C, while the end of the transformation is at 316 °C. The weight loss in this temperature range was 75%. Glycerol evaporates completely at a temperature above 290 °C. The change in the mass was simultaneously registered with the degradation of starch, and these two transformations overlap.

In the first stage, up to a temperature of about 473 K, there is a loss of mass (about 5–20%) as a result of evaporation of water contained in the structure of ion exchangers. At temperatures above 473 K, there is a loss of weight related to the distribution of functional groups. The weight loss from 600 K to 1200 K was gradual, which may have been due to the slow degradation of the styrene-divinylbenzene matrix. Lewatit SP112 TG and TG/TGA curves were also presented in [46], and Amberlite IRC 747 and Amberlite IRC 748, as well as Lewatit TP 208, in [49]. 

#### 3.1.3. XRD Analysis 

Regarding the other methods, X-ray powder diffraction (XRD), used to distinguish the crystalline phase, and gel permeation chromatography (GPC), used to determine the molecular weight of the polymers, are mainly applied. Native starch has a partially crystalline structure, and this phase accounts for approx. 45% of the total structure, the rest is the amorphous phase. The crystal structure of native starch is destroyed during plasticization. However, for the samples with glycerol, the crystalline phase content was 25% [39]. As for the GPC method, it was found that the average molecular weight of native starch is approx. 33 MDa and TPS is 3.52 MDa. After the application of modifiers, this mass increased and amounted to 39 MDa. Due to the polymer structure, SP112, S940, IRC 747, IRC748, IRC718, TP207, TP208, and S930 were not characterized by either XRD and GPC methods.

#### 3.1.4. ASAP ANALYSIS 

Characterization of the porous structure parameters of the selected sorbents and ion exchangers, i.e., the specific surface area S_BET_ (m^2^/g), pore size, and pore volume, was determined using low-temperature nitrogen adsorption/desorption isotherms obtained by means of an ASAP 2405 sorption analyzer (Micromeritics, Norcross, GA, USA) and are presented in [46]. The exemplary results are also presented in Table 5.

IRC748 was characterized by the highest S_BET_ value (23.1 m^2^/g) and TP 208 the lowest value (0.68 m^2^/g). The obtained pore size values indicate the presence of mesopores (range 2–50 nm) and macropores (>50 nm) in the structure of the ion exchangers. Such an extensive network of large pores inside the ion exchanger beads may allow the sorption process to proceed quickly.

#### 3.1.5. SEM Analysis

**Figure 7:**
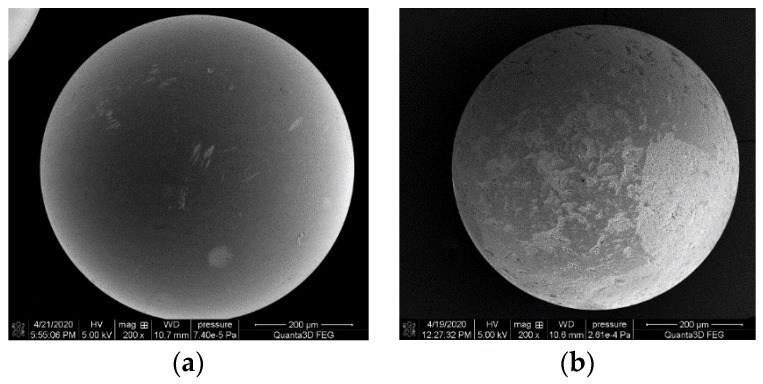

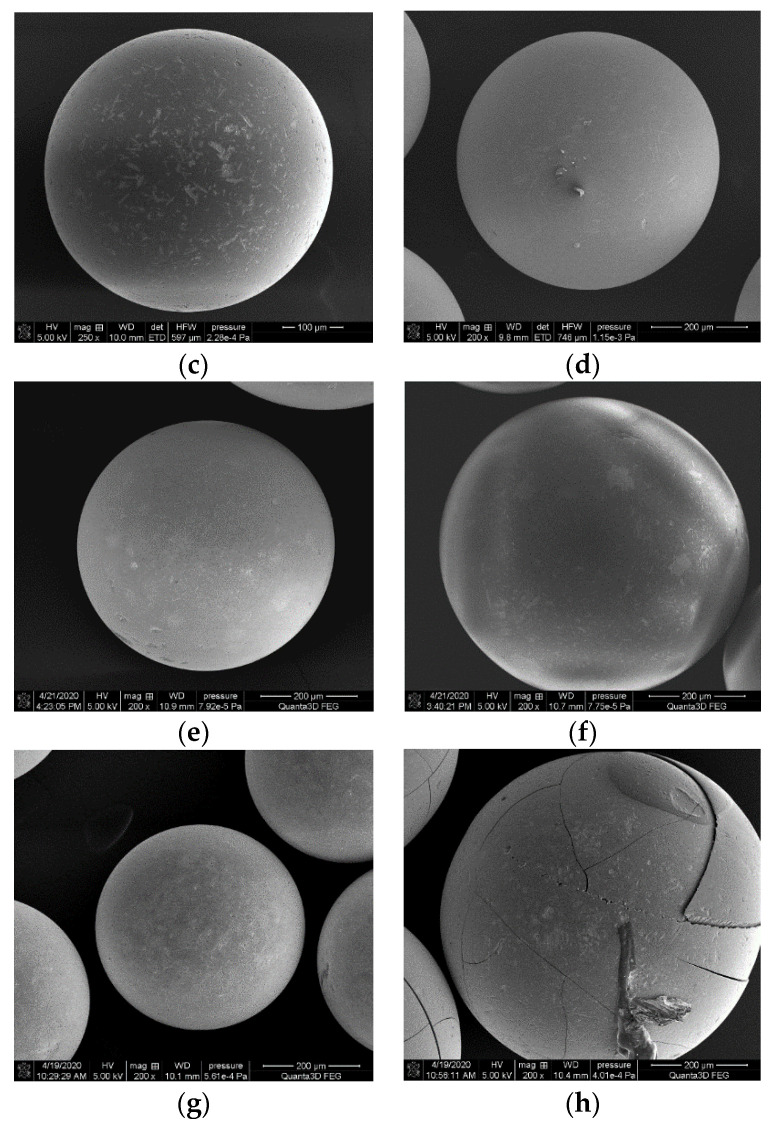
SEM micrographs of (**a**) SP112, (**b**) S940, (**c**) IRC 747, (**d**) IRC 748, (**e**) IRC 718, (**f**) TP207, (**g**) TP 208, and (**h**) S930.

SEM analysis of the materials revealed that, as mentioned in the table presenting the physicochemical properties, ion exchangers differ in bead size (presented images are in the same magnification). Their surface is typical of macroporous materials (Figure 7). Analogous results were presented in [49,50,51]. 

#### 3.1.6. pHpzc Analysis

**Figure 8:**
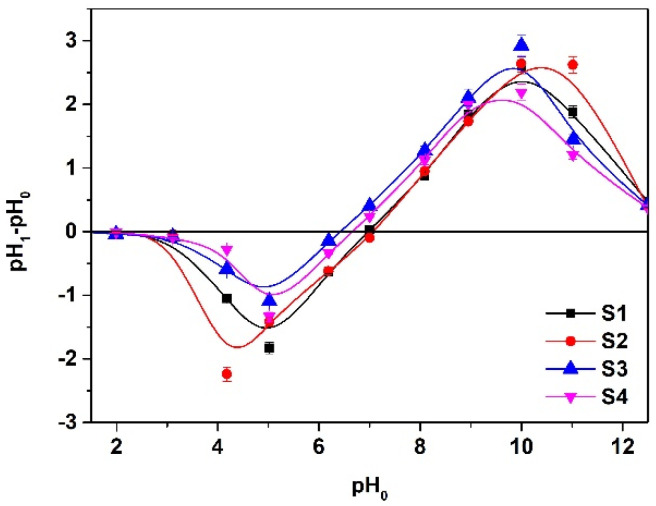
Determination of point of zero charge for S1–S4 by the drift method. Analogous values for the ion exchangers are as follows: 6.61 (SP112), 9.98 (S940), (IRC 747), (IRC748), (IRC718), (TP207), (TP208), and 6.67 (S930).

The pH of zero charge (pH_pzc_) of S1–S4 sorbents was determined using the solid addition method. The solutions of 0.01 M KNO_3_ concentration of proper pH, which was in the range from 1 to 12, were prepared using 0.1 M HCl and 0.1 M NaOH. Then 0.2 g of sorbent was brought into contact with 20 cm^3^ of solution for 24 h (vibration amplitude 8 units, shaking speed 180 rpm). After this period of time, the pH of the solution was measured again (pH_1_) using a pH-meter CP-411 (Elmetron, Zabrze, Poland). The plot pH_0_ vs. pH_1_-pH_0_ was obtained and is presented in Figure 8. The values of the point of zero charge were equal to 6.42 (S3), 6.66 (S4), 7.04 (S1), and 7.05 (S2).

#### 3.1.7. Degradation Analysis

Our previous studies showed that thermoplastic blends of starch with, among others, glycerol, poly (butylene succinate), and polylactic acid have a good potential to be used in heavy metal ion removal [52]. In this work, the thermal behavior of these materials was investigated by differential scanning calorimetry (DSC), thermogravimetric analysis (TGA), and Fourier-transform infrared (FTIR) spectroscopy. It was proven that chemical interactions between the different components occur and degradation mechanisms were identified, being assigned to the mass loss due to the plasticizer leaching and to the degradation of the starch at temperature above 350 °C. For biodegradation to be visible and occur quickly, the starch content in the material must be at least 60%. In such cases, the starch is a matrix of the material, and this is usually plasticized starch. The higher the starch content, the more biodegradable the polymer [52].

### 3.2. Influence of Solution Ph on the Uptake of Cd(II) Ions

**Figure 9:**
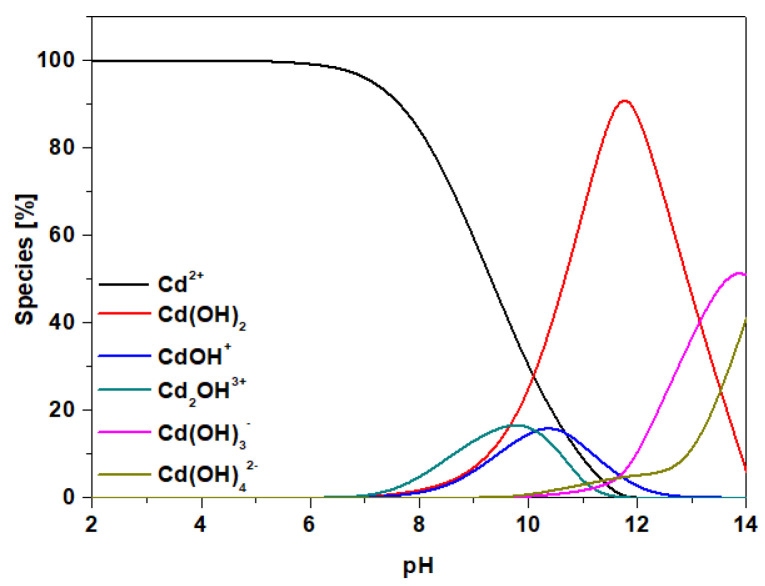
Form of Cd(II) species depending on pH values at the initial concentration 100 mg/dm^3^.

Due to the surface properties of adsorbents, one of the factors that is taken into account when assessing their adsorption capacity is pH. Its influence is related to both the effect on the chemical behavior of the adsorbent, as well as the adsorbate, i.e., the species present in the solutions at different pH values and the nature of the structures formed between the adsorbate and the adsorbent. According to literature data on the speciation of cadmium ions, the following forms were found to be the dominant species: Cd^2+^ and Cd(OH)^+^ at pH <8 and Cd(OH)_2_ at pH >8 at the initial concentration 100 mg/dm^3^ (Figure 9) [53].

**Figure 10:**
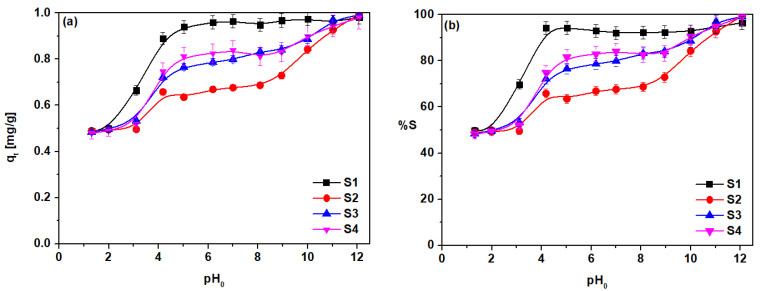
Influence of solution pH on Cd(II) sorption on S1–S4 (**a**) *q_t_* and (**b**) %S (*c*_0_ = 10 mg/dm^3^ Cd(II), *A* = 8, 180 rpm, 293 K, *m* = 0.2 g, *V* = 20cm^3^, *t* = 4 h).

This is of particular importance in the case of the ion exchangers with the chelating functional groups. The hydronium H_3_O^+^ ions compete for active sites on the sorbent surface with metal cations, especially at low pHs of the solution; therefore, usually low adsorption is observed in this area. The adsorption of metal ions increases in the pH region when decreasing the number of hydronium ions. This is due to the fact that the surface of the adsorbent becomes negatively charged as a result of the deprotonation reaction. Accordingly, the repulsive force that exists between the metal ions in the solution and the active groups of sorbents is reduced, thereby increasing the removal of metal ions from the solution. Therefore the Cd(II) ion sorption mechanism is largely based on the ion exchange process between the exchangeable protons from the sorbents hydroxyl groups and the metal ions, or a complexation reaction in the case of SP112, S940, IRC747, IRC748, IRC718, TP207, TP207, and TP208, as well as S930. This was confirmed by the fact that the equilibrium pH of the solutions was reduced compared to the initial pH. This can be expressed in the following reactions: Cd^2+^ + H_2_O ⇄ Cd(OH)^+^ + H^+^ and Cd(OH)^+^ + H_2_O ⇄ Cd(OH)_2_ + H^+^. The influence of solution pH on Cd(II) sorption using the S1–S4 sorbents is presented in Figure 10.

The highest adsorption, close to 1 mg/g, was found for the sorbent S1, at pH 4–12 there was a plateau at a level of 95% adsorption. In the case of sorbents S2–S4, a sinusoidal course of the adsorption change, depending on pH, was observed. In the range of pH 0–2 the increase in adsorption for each of the three sorbents was found to be at least 50% sorption, and the sorption capacity was 0.5 mg/g for S2–S4. Another increase in sorption was found in the range of pH 3–4 and it was up to 0.89 mg/g for S1, 0.66 mg/g for S2, 0.72 mg/g for S3, and 0.75 mg/g for S4. In the pH range from 6–8 they were equal to 0.96 mg/g for S1, 0.67 mg/g for S2, 0.79 mg/g for S3, and 0.82 mg/g for S4. Another increase in adsorption took place in the range of pH 9–11, to the level of about 98%, and the sorption capacity was almost 1 mg/g. In this pH range, the precipitation of Cd(OH)_2_ hydroxide on the adsorbent surface, which results in a high adsorption value, was observed. As the optimum, pH 6.0 was chosen for further experiments.

### 3.3. Sorption Process

**Figure 11:**
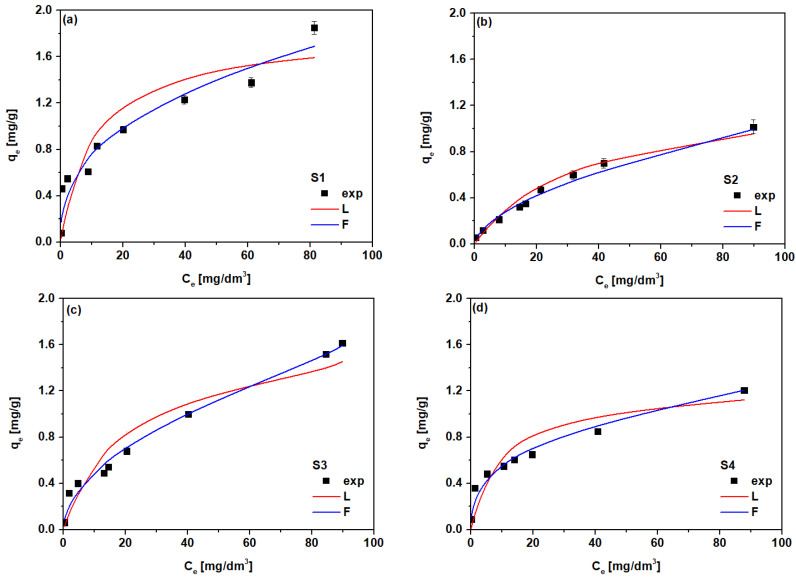
Adsorption isotherms of Cd(II) for (**a**) S1, (**b**) S2, (**c**) S3 and (**d**) S4 and fitting of the experimental data to the Freundlich and Langmuir isotherms models.

The main source of heavy metal pollution in China is industrialization. In rural areas the Cd(II) concentration in the atmosphere is lower than 1.0 pg/dm^3^; reaching a value up to 100 pg/dm^3^ in urbanized areas. Cd(II) can be precipitated and accumulated in the soil [54]. Metal uptake by plants occurs during the adsorption of soil solution by roots and then their transport by the xylem network to the leaves. On the other hand, transpiration establishes a water potential gradient in the plant and the excess of heavy metals affects water flow efficiency by reducing the transpiration rate and/or through changes in stomal resistance in leaves. Hydraulic conductivity influences the water supply for the whole plant, so that the water transport, root exudation, and leaf gas exchange parameters depend on the toxic metal concentrations. These plant–water relationships impair the shoot water and have a negative effect on the plant growth, largely causing the plant to develop negatively. For instance, a high concentration of Ni(II) in the root zone of *Psidium guajava* can rapidly inhibit stomatal aperture (>50%) [55]. Particularly high levels of Hg(II) ions, which bind to water channel proteins, cause leaf stomatal closure and physical obstruction of water transport, and they further induce biomembrane lipids and cellular metabolism disruption. Hg(II) uptake by *Brassica juncea* L. can cause phytotoxicity of both biomass and leaf, as regards the water content reduction and leaf cellular structure changes, which are similar to those of *Pteris vittata* under Cr(VI) stress [56,57]. When the heavy metal ions are centripetally transported to the vascular cylinder, they are loaded into xylem conducts, where they are further transferred to the aboveground parts of the plant, including stems, leaves, blossoms, fruits, and seeds. Therefore, in our studies the extract based on *D. officinale* in different forms (stick and balls), which is commonly used in Chinese herbal medicine, was prepared for the Cd(II) ion removal determination. As a first step, adsorption studies were carried out. The effects of the initial concentration of Cd(II) on the sorption process with TPS based sorbents S1–S4 were investigated to determine the maximum sorption capacity. The results are presented in Figure 11.

To describe the interactions between the Cd(II) ions and the above mentioned adsorbents, the most popular adsorption isotherms were applied to fit the experimental data to the Freundlich and Langmuir models. The Freundlich and Langmuir isotherm parameters were calculated from the linearized plots of log *q_e_* vs. log *C_e_* and *C_e_*/*q_e_* vs. *C_e_*, respectively. Moreover, the obtained results were described using the 1/*n* parameter and the separation factor *R_L_* (Table S3). The Freundlich model is based on a different approach, it does not predict surface saturation and considers the existence of a multilayered structure. The Langmuir isotherm model predicts the formation of an adsorbate monolayer on the homogeneous adsorbent surface, with no side interactions between the adsorbed ions. The calculated isotherm parameters for the Cd(II) on S1–S4 are presented in Table 6.

For comparison, the same data were compiled for Cd(II) using the ion exchangers SP112, S940, IRC 747, IRC748, IRC718, TP207, TP208, and S930 and are shown in Table S4. The correlation coefficients R^2^ of the Freundlich model, equal to 0.909–0.986, were slightly higher than these of the Langmuir model, 0.857–0.944 for Cd(II) sorption using the S1–S4 sorbents. The 1/*n* values were in the range 0.377–0.573, which indicates the preferential adsorption of Cd(II). The adsorption capacities decreased for the sorbents modified by PBS and PLA. The Freundlich adsorption capacity k_F_ was equal to 0.323 mg/g for S1, 0.075 mg/g for S2, 0.148 mg/g for S3, and 0.243 mg/g for S4. The highest value for *k_F_* was in the case of S4 sorbent with the composition of 50%TPS + 50%PLA with 5% hemp fibers. 

The Langmuir isotherm is frequently used for the quantitative comparison of different adsorbents. The monolayer adsorption capacities were in the range of 1.26–1.79 mg/g with R^2^ 0.857–0.944, the highest *Q*_0_ value was found for S3. The calculated values of the separation factor *R_L_* e.g., 0.092–0.257 were in the range from 0 to 1, which proves the favorable nature of adsorption. Table 7 reports a comparison of the sorption capacities of starch based adsorbents for Cd(II) ions reported in the literature.

The applicability of the Langmuir isotherm model for description of the equilibrium sorption data of Cd(II) on ion exchangers was obtained regarding the R^2^ values and is presented in Table S4. Taking into account the values of monolayer capacities from 83.16 mg/g to 171.78 mg/g in relation to Cd(II), the following series of applicability can be noted, considering their application for the removal of Cd(II): IRC748 > TP208 > SP112 > TP207 > S930 > IRC718 ≈ S940 > IRC747.

### 3.4. Kinetic Studies

**Figure 12:**
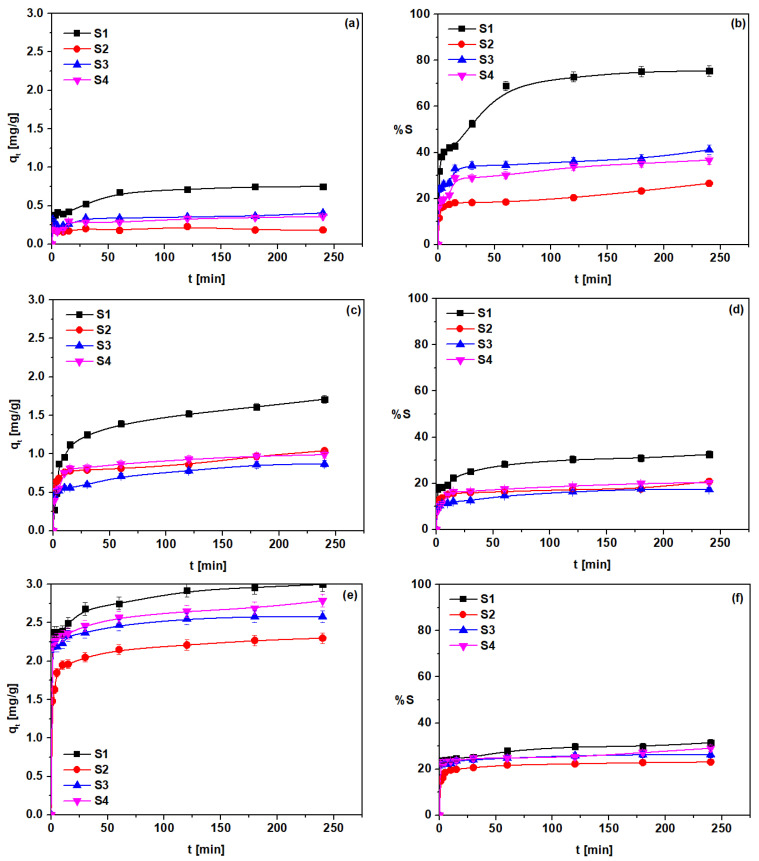
Amount of Cd(II) adsorbed on S1–S4 at the initial concentrations (**a**,**b**) 10 mg/dm^3^, (**c**,**d**) 50 mg/dm^3^, and (**e**,**f**) 100 mg/dm^3^.

The time-dependent behavior of Cd(II) sorption with S1–S4 was investigated in the range from 1 min to 4 h, and the amount of Cd(II) adsorbed (*q_t_*) on S1–S4 as well as the sorption percent (%*S*) were calculated. The plots *q_t_* vs. *t* are presented in Figure 12a,c,e. The plots %*S* vs. *t* are presented in Figure 12b,d,f. Additional curves are presented in Figure S2 (Supplementary Materials).

The initial concentrations of Cd(II) in the extract from *D. officinale* were 10, 50, and 100 mg/dm^3^, whereas the pH was close to 6. It was observed that the sorbents S1–S4 possessed different abilities to remove Cd(II) from the extract. The amount of Cd(II) adsorbed with S2 increased sharply within the first 60 min of phase contact time, then the system reached the equilibrium after 120–240 min, depending on the initial Cd(II) concentration in the extract. For the other (S1, S3, and S4) sorbents, the adsorption at the beginning was not so fast; therefore, the shape of the curve is not as sharp as in the case of S2. Thus, the amount of adsorbed Cd(II) increases slightly as the phase contact time increases. This indicates that the surface of the studied sorbents possesses adsorption sites which are occupied during the adsorption process, and the rate of adsorption decreases with the decrease of the number of empty adsorption sites. 

Moreover, the sorption capacities increase when the initial concentration increases for S1–S4 sorbents, being 0.36 mg/g (at the initial concentration 10 mg/dm^3^), 0.99 mg/g (at initial concentration 50 mg/dm^3^), and 2.79 mg/g (at initial concentration 100 mg/dm^3^); whereas for S3 they were as follows: 0.41 mg/g, 0.87 mg/g, and 2.58 mg/g. The adsorption capacities for S2–S4 were smaller compared to those of S1, e.g., 2.31 mg/g for S2, 2.58 mg/g for S3, and 2.79 mg/g for S4, and 3.01 mg/g for S1 (adsorption from extract containing 100 mg Cd(II)/dm^3^). 

The greatest adsorption capacities obtained with the S1 sorbent (TPS containing 30% of glycerol and 8% of water) indicate a strong affinity of Cd(II) for this sorbent. On the other hand, TPS adsorbents with PLA (50% of PLA and 50% of PLA with 5% hemp fibers (S3 and S4)) and with PBS (75% of BPBS (S2)) additions showed a smaller adsorption affinity for Cd(II). For Cd(II) sorption from *D. officinale* solutions of 10 mg Cd(II)/dm^3^, the adsorption capacities decreased from 0.75 mg/g for S1, through 0.41 mg/g for S3, to 0.36 mg/g for S4. Based on the adsorption capacities, the S1–S4 adsorbents can be ordered in the affinity series: S1 > S3 > S4 > S2 (adsorption from extract containing 10 mg Cd(II)/dm^3^), S1 > S2 > S4 > S3 (adsorption from extract containing 50 mg Cd(II)/dm^3^), as well as S1 > S4 > S3 > S2 (adsorption from extract containing 100 mg Cd(II)/dm^3^). 

A plasticizer (glycerol, sorbitol, glycols, urea, maltodextrin, water) is a material incorporated into a plastic material, resulting in its flexibility and increase in applicability. Starch granules are penetrated by plasticizer, and the inner hydrogen bonds of starch are broken at a high temperature or pressure. Moreover, the starch network can be deformed without rupture, due to the more mobile and smaller molecules of plasticizers. Starch–starch interactions are eliminated and starch–plasticizer interactions are formed. The percentage removal of TPS sorbents indicates that the Cd(II) removal is not quantitative. The %S was the highest for the S1 sorbent, being in the range from 32 to 75% (S1), from 12 to 27% (S2), from 24 to 41% (S3), and from 16 to 37% (S4) (at initial concentration 10 mg Cd(II)/dm^3^); whereas for the extract containing 100 mg Cd(II)/dm^3^ %S was from 22 to 31% (S1), from 15 to 23% (S2), from 22 to 26% (S3), and from 22 to 29% (S4). Comparing the %S values obtained during Cd(II) adsorption from the *D. officinale* extract containing 10 mg/L with the %S values obtained during Cd(II) adsorption from the extract with the higher Cd(II) concentration, 50 mg/dm^3^ or 100 mg/dm^3^, it was revealed that the %S was higher for the solutions with a smaller Cd(II) concentration. 

**Figure 13:**
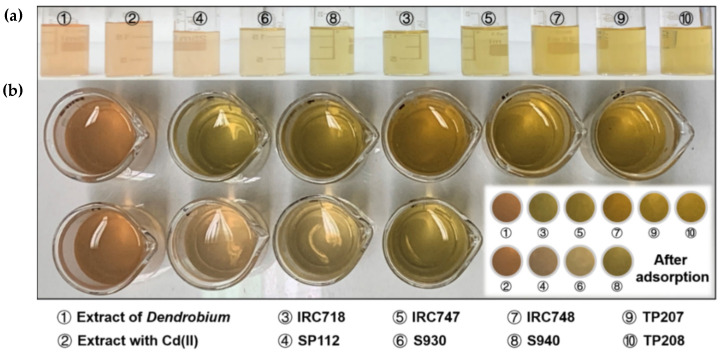
*D. officinale* extracts (**a**) before and (**b**) after adsorption on SP112, S940, IRC 718, IRC 747, IRC 748, TP207, TP 208, and S930.

The effects of phase contact time on the Cd(II) adsorption from *D. officinale* extracts containing 10, 25, and 50 mg/dm^3^ of Cd(II) and %S were also analyzed for chelating ion exchangers SP112, S940, IRC 718, IRC 747, IRC 748, TP207, TP 208, and S930. The kinetic curves were plotted as *q_t_* vs. *t* and %S vs. *t*. The exemplary results are presented in Table S4 (Supplementary Materials). Additionally, the *D. officinale* extracts before and after adsorption the ion exchangers are presented in Figure 13.

Based on the obtained results it was found that the amount of Cd(II) adsorbed on SP112, S930, S940, IRC718, and IRC747 increased very sharply at the beginning of the sorption process, within first 15–30 min of phase contact time for all systems under discussion. The results demonstrate that the systems reached equilibrium after 30 min in the case of the extract containing 10 mg Cd(II)/dm^3^, whereas for solutions of higher Cd(II) concentration this time was longer, being in the range 30–60 min (adsorption from the extract containing 25 mg Cd(II)/dm^3^) or 60–120 min (adsorption from the extract containing 50 mg Cd(II)/dm^3^). In the case of IRC748, TP207, and TP208, the kinetic curves rose gradually and the q_t_ increase was slower. The time required to reach system equilibrium was longer for IRC748, TP207, and TP208 compared to the SP112, S930, S940, IRC718, and IRC747 ion exchangers and increased with a Cd(II) initial concentration increase (e.g., 120 min for IRC748 and 10 mg/dm^3^; 240 min for IRC748 and 25 mg/dm^3^; >240 min for IRC748 and 50 mg/dm^3^, or >240 min for TP207 and TP208 and 10, 25, and 50 mg/dm^3^). The highest sorption capacities were obtained at 10 mg/dm^3^ for IRC748 and IRC747 (1.28 mg/g and 1.38 mg/g, respectively), at 20 mg/dm^3^ (2.21 mg/g and 2.79 mg/g), at 25 mg/dm^3^ (4.93 mg/g) for IRC748, and at 50 mg/dm^3^ (5.11 mg/g) for IRC747, whereas the smallest were for S957 (0.09 mg/g, 10 mg/dm^3^), IRC718 (1.64 mg/g, 25 mg/dm^3^), TP207 (2.55 mg/g, 50 mg/dm^3^) ion exchangers. The results show that ion exchangers of uniform bead size distributions (0.52–0.66 mm IRC748, 0.50–0.65 mm IRC747) allowed obtaining the largest Cd(II) uptake. Fine resin beads provide a greater capacity and better kinetics, whereas coarse beads are often more sensitive to osmotic stress and have slower kinetics, resulting smaller in adsorption efficiency. The adsorption process of Cd(II) on ion exchangers as a heterogeneous reaction between the solid phase (ion exchanger) and the aqueous phase (extract containing Cd(II)) is a complex and multistage process, in which individual stages determine the overall rate of Cd(II) adsorption: (1) transport of Cd(II) to the external surface of ion exchange resin; (2) diffusion of Cd(II) ions through the ‘liquid film’ surrounding the ion exchanger beads, diffusion through film (film diffusion model); (3) migration of Cd(II) ions within ion exchange beads, so-called intraparticle diffusion in ion exchanger pores; (4) direct adsorption of Cd(II) ions on binding functional groups of the ion exchanger (considered as a chemical reaction); (5) diffusion of exchange ions within the ion exchanger beads (reversal of stage 3); and (6) diffusion of exchange ions from the ion exchanger into the solution (reversal of stage 2). Film diffusion is usually the rate-controlling step for systems with a small particle size, poor mixing, small concentration of adsorbate, and high affinity of the adsorbate for the adsorbent; whereas the intraparticle diffusion for systems with large particle sizes of the adsorbent gives a low affinity of adsorbate for the adsorbent, good mixing, and high concentration of the adsorbate [62]. %S of Cd(II) was much higher for the ion exchangers than for the TPS sorbents. The purification efficiency of the extracts containing 10, 25, and 50 mg/dm^3^ Cd(II) was great, and the %S was in the range from 84.2 to 98.71% for SP112, from 64.65 to 97.33% for S930, from 78.79 to 97.90% for S940, from 26.61 to 99.71% for S957, from 83.62 to 97.16% for IRC718, from 80.71 to 97.73% for IRC474, from 27.5 to 94.72% for IRC748, from 20.07 to 93.06% for TP207, and from 25.19 to 82.19% for TP208. Comparing these results with those described in the literature it can be noted that the adsorption of heavy metal ions with Cd(II) on Amberlite IR120 is efficient, with an %S equal to 99% and pH 4–8 and after 75 min for Cd(II). The maximum adsorption capacity was equal to 1.78 mg/g for Cd(II) [63]. Application of Dowex 50W × 8 for Cd(II) and other heavy metals (Cu(II), Zn(II), Ni(II), Pb(II)) also shows fast kinetics. In the case of Cd(II) the system reached equilibrium after 60 min. At pH 8–9, %S 97% of Cd(II) was removed [64]. Moreover, the results obtained for Duolite C443 and Lewatit CNP80 demonstrate that the adsorption increased with the increasing phase contact time, and after 75 min the system reached equilibrium for Cd(II) [65]. 

**Figure 14:**
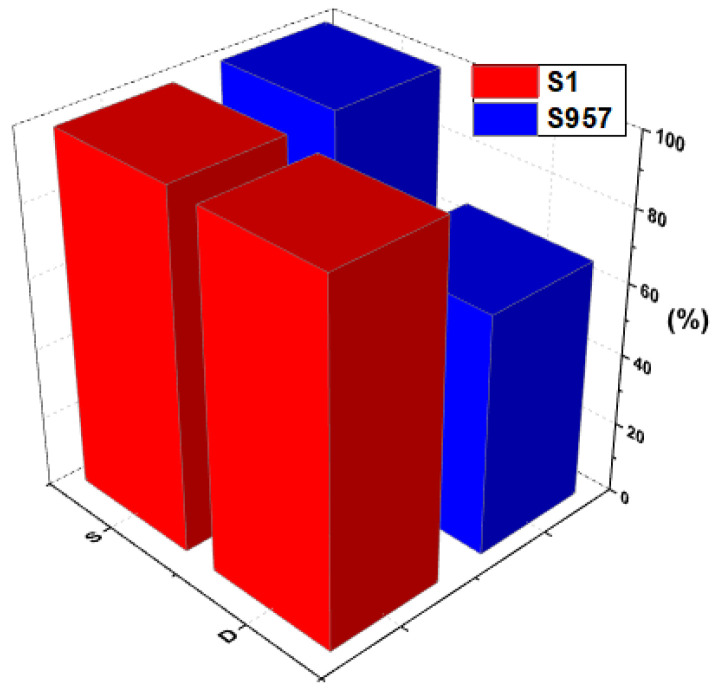
Comparison of sorption–desorption of Cd(II) on S1 and S957 at initial concentration of 10 mg/dm^3^.

In the next step, desorption studies were also carried out using hydrochloric acid as eluent. Elution involves removing the Cd(II) from the used materials by reversing the adsorption process. The main factor that makes desorption a simple process is the fact that cadmium will only adsorb onto the surface of TPS-based materials. Other requirements of the elution process are high HCl concentration, low ionic strength of solution, optimum flow rate/speed of mixing, and low cadmium concentration in the solution. A comparison of Cd(II) elution efficiency using 1 M HCl from the best S1 sorbent and ion exchanger S957 is presented in Figure 14. In the case of 1 M HCl, the desorption was almost completed (100%) for S1 sorbent; however, in the case of S957 it was equal to 67%. In the case of the low-cost adsorbents (like S1–S4), their rare reuse was taken into account, due to the fact that the amount of the cadmium(II) adsorbed is not high. Therefore, disposal of the exhausted adsorbent is proposed. The obtained results also suggest the different mechanisms of sorption: for the TPS based sorbent, physical adsorption, and for IXs, chemical reaction. 

## 4. Conclusions

For purification of *D. officinale* extracts thermoplastic starch (S1); modified TPS with poly (butylene succinate), 25% of TPS + 75%PBS (S2); 50% of TPS + 50% PLA (S3); and 50% of TPS + 50% PLA with 5% of hemp fibers (S4); as well as ion exchangers of different types, e.g., Lewatit SP112, Purolite S940, Amberlite IRC747, Amberlite IRC748, Amberlite IRC718, Lewatit TP207, Lewatit TP208, and Purolite S930 were used. The effects of adsorption time, initial concentration of Cd(II), and pH on the adsorption efficiency were investigated. The results showed that modifications of TPS do not improve the adsorption capacity of Cd(II). The adsorption efficiency of Cd(II) on S1 is over 99% with the adsorption capacity of 1.79 mg/g, and on IRC 748 with the adsorption capacity of 171.78 mg/g. The adsorption rate of Cd(II) on the S1–S4 sorbents and ion exchangers SP112, S940, IRC 718, IRC 747, IRC 748, TP207, TP 208, and S930 was determined from the PSO kinetic equation. The adsorption efficiency of ion exchangers was higher than on S1–S4 for Cd(II), which indicates that they can be used for utilization of Cd(II). This research proposes novel materials based on TPS, to be used to deal with the cadmium contamination of medicine herbal extracts, as well as providing a highly efficient adsorbent for further applications. The results obtained clearly show their advantages for use as natural, cheap, and abundant materials.

## Figures and Tables

**Table 1:** Some transporters involved in heavy metal accumulation in plants.

Names	Functions Related to Heavy Metal Accumulation	Transporter Types	Reference
**ZIPs family transporters (Zn, Fe, Mn, Cd, Ni, Cu, etc.)**			
ZIP1	Transporting Cd to the cytosol	Divalent cations exporter transporter	[16]
ZIP2	Transporting Cd(II)/Zn(II) to the root vascular system	Plasma membrane -localized transporter	[17]
NcZNT1	Zn(II)/Cd(II) long-distance transport of vascular system	Plasma membrane -localized transporter	[18]
AtZIP9	Marker of Zn(II) deficiency or Cd(II) excess in uptake system	Metal transporter	[19]
**CDFs family transporters (Mn, Fe, Zn, Co, Cd, Ni, etc.)**			
MTP1	Transporting cytosolic Zn/Cd into vacuoles	Vacuolar transporter	[20]
MTP4	Participating in vacuolar Zn and Cd sequestration	Vacuolar transporter	[21]
**NRAMPs family transporters (Mn, Fe, Zn, Cd, Co, Ni, Pb, etc.)**			
AtNRAMP1	High-affinity transporting Mn(II) into root cells; Implicating in Cd(II) uptake in endodermal cells	Plasma membrane-localized transporter	[22]
AtNRAMP3/4	Exporting vacuolar Fe(II)/Mn(II)/Cd(II) into the cytosol	Metal transporters	[23]
NcNRAMP1	Transporting Cd(II) into endodermal cells; Involving Cd(II) flux movement towards the stele and root-to-shoot Cd transport	Cd hyperaccumulation transporter	[24]
**HMAs family transporters (Cu, Ag, Zn, Cd, Pb, etc.)**			
AtHMA1	Exporting excessive Cd(II), Cu(II), Zn(II) from the chloroplast to the cytosol	Chloroplast-localized transporter	[25]
AtHMA2	Exporting of cytosolic Zn(II) and Cd(II) into the vascular cylinder	Plasma-membrane-localized transporters	[26]
AtHMA3	Transporting cytosolic Co(II), Zn(II), Cd(II) and Pb(II) into vacuoles	Metal transporter	[27]
AtHMA4	Transporting cytoplasmic Zn(II), Cd(II), Co(II) to the xylem vessels	Plasma-membrane-localized transporters	[28]
**ABCC sub-family transporters (As, Cd, Hg, etc.)**			
DK/2	Transporting Cd-PCs and Hg-PCs into vacuoles	Vacuolar phytochelatin transporters	[29]
**CAXs (Cation/H^+^ antiporters) family (Mn, Zn, Cd, Ni, Cu, etc.)**			
CAX2	Transporting Mn(II), Zn(II) and Cd(II) into the vacuole	Vacuolar transporter	[30]
AtCAX4	Transporting Mn(II), Ni(II) and Cd(II) into the vacuole	Vacuolar transporter	[31]

**Table 2:** Thermoplastic starch used for Cd(II) removal from the *D. officinale* extracts.

Name	Appearance	Composition	Name	Appearance	Composition
S1	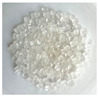	TPS (containing starch + 30% glycerol + 8% H_2_O)	S3	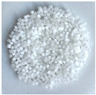	50% of TPS + 50% of PLA
S2	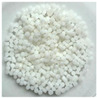	25% of TPS + 75% of PBS	S4	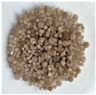	50% of TPS + 50% of PLA (5% hemp fibers)

**Table 3:** Ion exchangers used for Cd(II) removal from the *D. officinale* extracts.

Name	Appearance	Composition	Name	Appearance	Composition
SP112	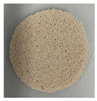	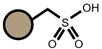	IRC718	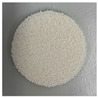	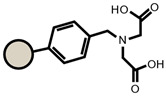
S940	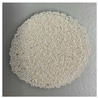	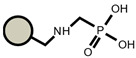	TP207	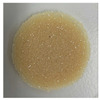	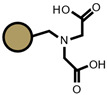
IRC747	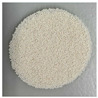	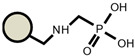	TP208	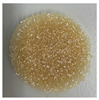	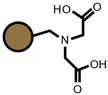
IRC748	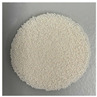	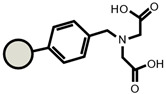	S930	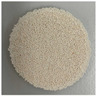	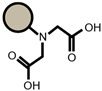

**Table 4:** Changes in the position of the characteristic bands in the spectra of S1–S2 sorbents.

Sorbent	Hydrogen Bonds	C-O in C-O-H	C-O in C-O-C
**S1**	3287 cm^−1^	1158 cm^−1^ and 1059 cm^−1^	1014 cm^−1^ and 996 cm^−1^
**S2**	3329 cm^−1^	1207 cm^−1^ and 1053 cm^−1^	1020 cm^−1^ and 994 cm^−1^
**S3**	3319 cm^−1^	1183 cm^−1^ and 1082 cm^−1^	1021 cm^−1^ and 998 cm^−1^
**S4**	3319 cm^−1^	1206 cm^−1^ and 1083 cm^−1^	1018 cm^−1^ and 990 cm^−1^

**Table 5:** Textural properties of S1.

S_BET_ [m^2^/g]	14.98
V_tot_ [cm^3^/g]	0.144

**Table 6:** Freundlich and Langmuir isotherm parameters determined for Cd(II) sorption in the S1–S4 systems using linear regression.

Sorbent	Freundlich Isotherm Parameters			Langmuir Isotherm Parameters			
	*k_F_* (mg/g)	1/*n*	R^2^	*Q*_0_ (mg/g)	*b* (dm^3^/mg)	*R_L_*	R^2^
S1	0.323	0.377	0.909	1.79	0.099	0.092	0.927
S2	0.075	0.573	0.986	1.32	0.029	0.257	0.857
S3	0.148	0.524	0.944	1.74	0.046	0.178	0.865
S4	0.243	0.358	0.967	1.26	0.097	0.094	0.944

**Table 7:** Comparison of the sorption capacities of starch based adsorbents for Cd(II) removal.

Adsorbent	*q_e_* (mg/g)	pH	Ref.
S1	1.79	6	This paper
S2	1.32	6	
S3	1.74	6	
S4	1.26	6	
Montmorillonite modified starch	4.2	5	[58]
Starch esters	7.54	4–9	[59]
Succinylated starch	12.36	4–7	
Magnetic starch microspheres	39.98	-	[60]
Amino (5.67–13.01 N%) modified starch	69.7–139.4	6–7	[61]

## Data Availability

The data presented in this study are available on request from the corresponding author.

## Supplementary Materials

The following are available online at https://www.mdpi.com/article/10.3390/ma14164734/s1, Figure S1: ATR-FTIR spectra of (a) native starch, (b) glycerol, (c) PBS, (d) PLA and (e)TPS, Figure S2: Kinetic curves for Cd(II) sorption on TP747, T207 and TP208; Table S1: PBS and PLA characterization, Table S2: Chelating ion exchange resins characteristics, Table S3: Description of kinetic and isotherm models, Table S4: Kinetic parameters obtained for TPS (S3) and ion exchangers (IRC748, TP207, TP208).
