# Supplementary material for: Medical Plant Extract Purification from Cadmium(II) Using Modified Thermoplastic Starch and Ion Exchangers
Source: Materials (Basel). 2021 Aug 22;14(16):4734. doi: 10.3390/ma14164734 (PMC8401945; doi:10.3390/ma14164734)
Supplement: Supplementary file 1 [file materials-14-04734-s001.zip › materials-1283557-supplementary.pdf]

materials

MDPI

Supplimentary materials

Medical Plant Extract Purification from Cadmium(II) Using Modified Thermoplastic Starch and Ion Exchangers

Yi-Gong Chen [1], Qian Wang [2], Anna Wołowicz [3], Agnieszka Gładysz-Płaska [3], Monika Wawrzkiewicz [3], Weronika Sofińska-Chmiel [4], Gui-Yuan Lv [5], Dorota Kołodyńska [3,*] and Su-Hong Chen [1,*]

[1] Collaborative Innovation Center of Yangtze River Delta Region Green Pharmaceuticals, Zhejiang University of Technology, Chaowang Road 18, 310014 Hangzhou, China; yigongchen@hotmail.com
[2] Hangzhou Fang Hui Chun Tang Group Co., Ltd., 310052 Hangzhou, China; wangq@fhct.com
[3] Department of Inorganic Chemistry, Institute of Chemical Sciences, Faculty of Chemistry, Maria Curie-Skłodowska University, M. Curie Skłodowska Sq. 2, 20-031 Lublin, Poland; anna.wolowicz@poczta.umcs.lublin.pl (A.W.); a.gladysz-plaska@poczta.umcs.lublin.pl (A.G.-P.); m.wawrzkiewicz@poczta.umcs.lublin.pl (M.W.)
[4] Analytical Laboratory, Institute of Chemical Sciences, Faculty of Chemistry, Maria Curie-Skłodowska University, M. Curie Skłodowska Sq. 2, 20-031 Lublin, Poland; wschmiel@poczta.umcs.lublin.pl
[5] College of Pharmaceutical Science, Zhejiang Chinese Medical University, 310053 Hangzhou, China; zjtcmlgy@163.com
* Correspondence: d.kolodynska@poczta.umcs.lublin.pl (D.K.); chensuhong@zjut.edu.cn (S.-H.C.)

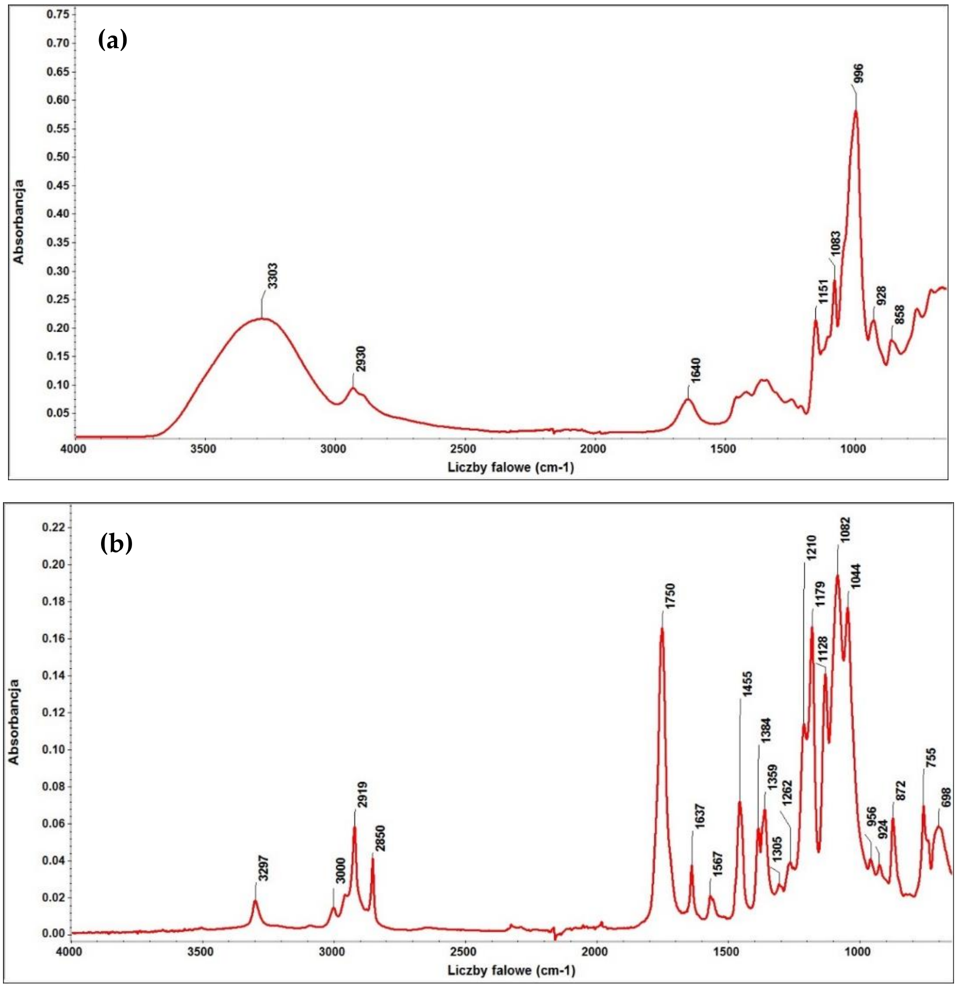

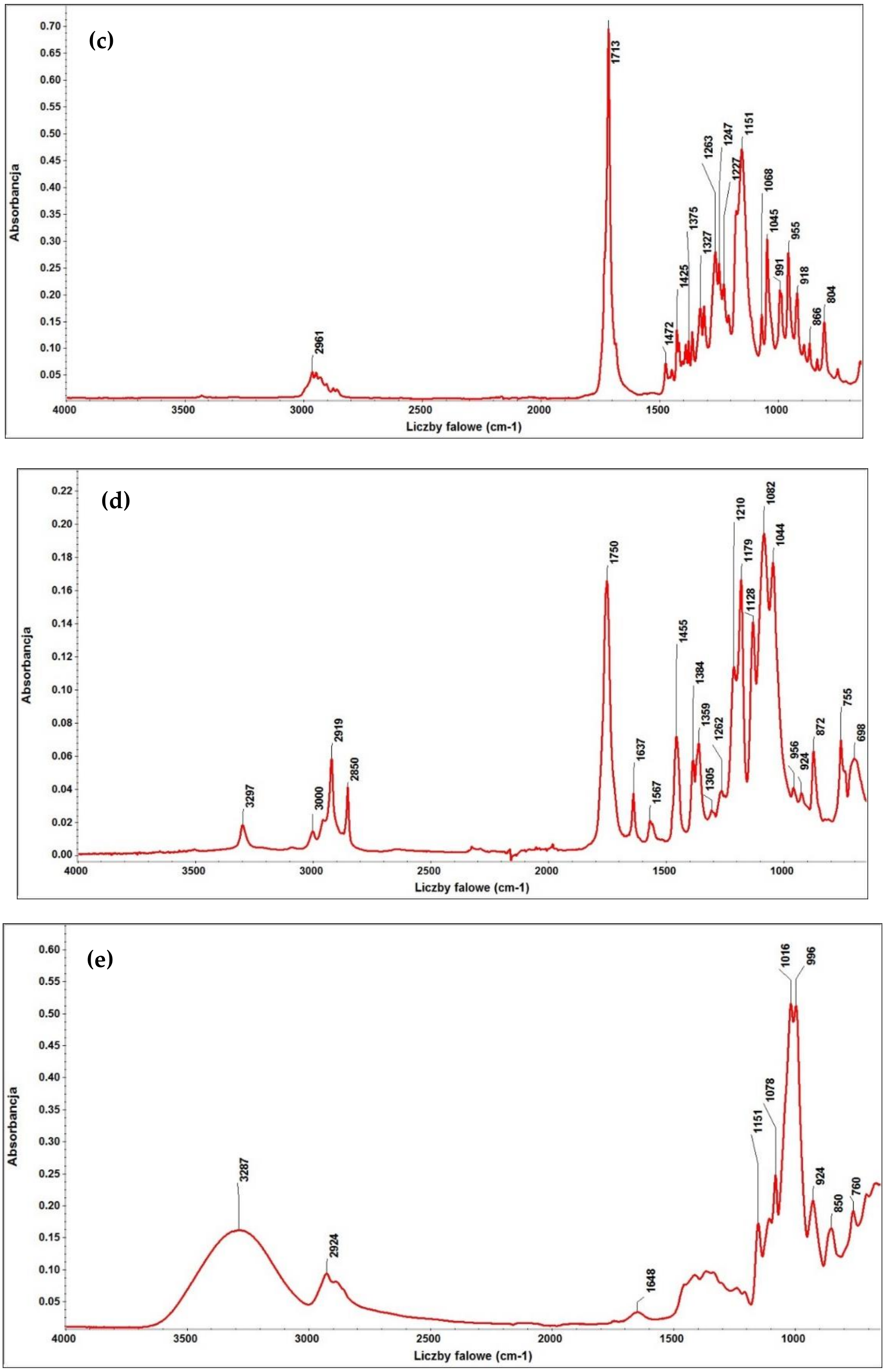

Figure S1. ATR-FTIR spectra of (**a**) native starch, (**b**) glycerol, (**c**) PBS, (**d**) PLA and (**e**) TPS.

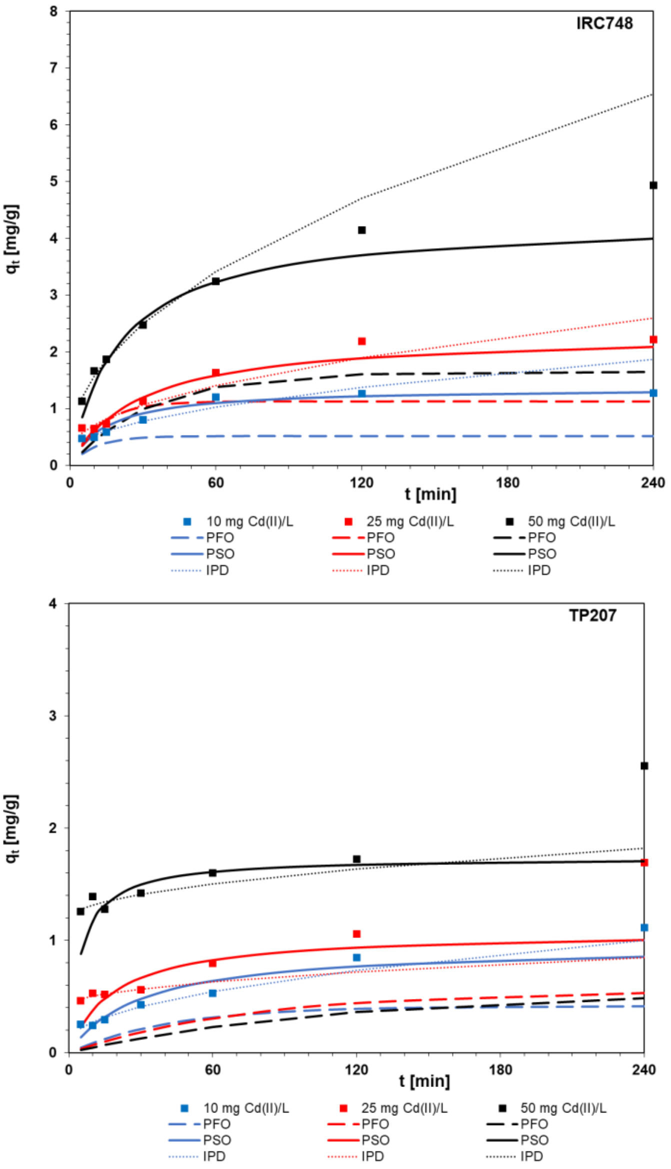
IRC748
q_t [mg/g]
t [min]
10 mg Cd(II)/L
PFO
PSO
IPD
25 mg Cd(II)/L
PFO
PSO
IPD
50 mg Cd(II)/L
PFO
PSO
IPD
TP207
q_t [mg/g]
t [min]
10 mg Cd(II)/L
PFO
PSO
IPD
25 mg Cd(II)/L
PFO
PSO
IPD
50 mg Cd(II)/L
PFO
PSO
IPD

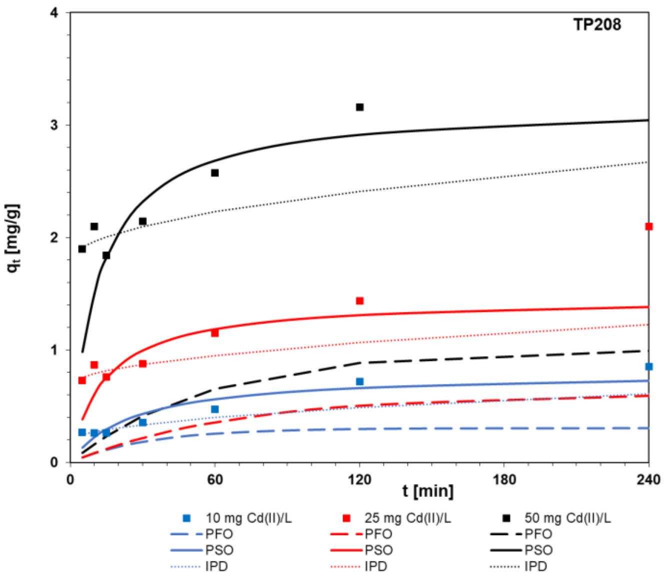

Figure S2. Kinetic curves for Cd(II) sorption on TP747, T207 and TP208.

Table S1. PBS and PLA characterization.

Parameters	PBS	PLA
Glass transition temperature [K]	241	328
Melting temperaturę [K]	387	413–453
HDT deflection temperature [K]	97	55
Tensile strength [MPa]	34	66
Elongation at break [%]	560	4
Crystallinity degree [%]	34–45	0–40

Table S2. Chelating ion exchange resins characteristics.

Name	Matrix	Mean bed size [mm]	Total capacity [eq/dm³]	pH range	Water retention [%]
**SP112**	PS-DVB	0.65 (±0.05)	1.7 (Na^+ form)	0–14	52–57 (Na+ form)
**S940**	PS-DVB	0.43–0.85	-	0–14	55–65 (Na^+ form)
**IRC747**	PS-DVB	0.52–0.66	≥ 1.75(Na^+ form)	0–14	64–69 (Na^+ form)
**IRC748**	PS-DVB	0.50–0.65	≥ 1.35 (Na^+ form)	1.5–14	60–65 (Na^+ form)
**IRC718**	PS-DVB	0.3–1.2	-	0–14	-
**TP207**	PS-DVB	0.4–1.25	2.42 (Na^+ form)	0–14	53–58 (Na^+ form)
**TP208**	PS-DVB	0.4–1.25	2.9 (Na^+ form)	0–14	55–60 (Na^+ form)
**S930**	PS-DVB	0.60–0.85	2.40 (Na^+ form)	2–6	55–65 (Na^+ form)

PS-DVB—polystyrene crosslinked with divinylbenzene.

Table S3. Description of kinetic and isotherm models.

Model	Equation	Parameters	Plot
		**Kinetic models**	

Pseudo-first order kinetic equation PFO	$\log(q_e - q_t) = \log q_e - \frac{k_1 t}{2.303}$	k_1 [1/min] is the rate constant of PFO equation<br>q_e [mg/g] is the adsorption capacity	log ($q_e - q_t$) vs. t<br>k_1 = -2.303 × slope<br>$q_e = 10^{intercept}$
Pseudo-second order kinetic equation PSO	$\frac{t}{q_t} = \frac{1}{k_2 q_e^2} + \frac{t}{q_e}$	k_2 [g/mg min) is the rate constant of PSO equation<br>q_e [mg/g] is the adsorption capacity<br>h [mg/g min] is the initial sorption rate	t/q_t vs. t<br>k_2 = slope^2/intercept<br>q_e = 1/slope<br>$h = k_2 q_e^2$
Intraparticle diffusion IPD	$q_t = k_i t^{0.5} + C$	k_i is the intraparticle diffusion rate constant [mg/g·min$^{0.5}$],<br>C in the constant illustrating the effect of the boundary layer on the sorption process	q_t vs. $t^{0.5}$
**Isotherm models**			
Freundlich	$\log q_e = \log k_F + \frac{1}{n}\log C_e$	k_F is the Freundlich adsorption capacity [mg/g]<br>$1/n$ is the Freundlich constant related to the surface heterogeneity	log q_e vs. log C_e<br>$k_F = 10^{intercept}$ [mg/g]<br>$1/n$ = slope
Langmuir	$\frac{C_e}{q_e} = \frac{1}{Q_0 b} + \frac{C_e}{q_0}$	Q_0 is the Langmuir monolayer sorption capacity [mg/g]<br>b is the Langmuir constant related to the free energy of sorption [dm^3/mg]<br>R_L is the separation factor or equilibrium parameter	C_e/q_e vs. C_e<br>Q_0 = 1/slope [mg/g]<br>b = slope/intercept [dm^3/mg]<br>$R_L = \frac{1}{(1 + b \times C_0)}$

Table S4. Kinetic parameters obtained for TPS (S3) and ion exchangers (IRC748, TP207, TP208).

System	$q_{e,exp}$ [mg/g]	PFO			PSO				IPD	
		q_e [mg/g]	k_1 [1/min]	R^2	q_e [mg/g]	k_2 [g/mg min]	h [mg/g min]	R^2	k_{int} [mg/g min$^{0.5}$]	R^2
**S3**										
10 mg/dm^3	0.75	0.60	0.041	0.879	0.76	0.184	0.11	0.998	0.030	0.649
25 mg/dm^3	1.71	0.74	0.009	0.888	1.52	0.138	0.32	0.999	0.066	0.862
50 mg/dm^3	2.75	0.42	0.014	0.456	2.95	0.109	0.95	0.998	0.047	0.402
**IRC748**										
10 mg/dm^3	1.28	1.19	0.042	0.987	1.36	0.050	0.09	0.996	0.108	0.941
25 mg/dm^3	2.21	2.59	0.036	0.945	2.34	0.015	0.08	0.978	0.152	0.952
50 mg/dm^3	4.93	3.80	0.013	0.996	4.33	0.011	0.21	0.988	0.403	0.985
**TP207**										
10 mg/dm^3	1.11	0.96	0.010	0.979	0.96	0.034	0.03	0.980	0.059	0.869
25 mg/dm^3	1.69	1.28	0.006	0.988	1.08	0.049	0.06	0.976	0.028	0.889
50 mg/dm^3	2.55	1.28	0.004	0.921	1.74	0.119	0.36	0.999	0.041	0.472
**TP208**										
10 mg/dm^3	0.85	0.70	0.013	0.967	0.80	0.048	0.03	0.981	0.028	0.725
25 mg/dm^3	2.10	1.41	0.006	0.975	1.46	0.048	0.10	0.986	0.035	0.821
50 mg/dm^3	4.07	2.32	0.008	0.972	3.18	0.028	0.28	0.989	0.057	0.281
